# Real-Time Map Matching with a Backtracking Particle Filter Using Geospatial Analysis

**DOI:** 10.3390/s22093289

**Published:** 2022-04-25

**Authors:** Dorian Harder, Hossein Shoushtari, Harald Sternberg

**Affiliations:** Geodesy and Geoinformatics, HafenCity Universität, 20457 Hamburg, Germany; hossein.shoushtari@hcu-hamburg.de (H.S.); harald.sternberg@hcu-hamburg.de (H.S.)

**Keywords:** map matching, particle filter, backtracking, correction, inertial odometry, geospatial analysis

## Abstract

Inertial odometry is a typical localization method that is widely and easily accessible in many devices. Pedestrian positioning can benefit from this approach based on inertial measurement unit (IMU) values embedded in smartphones. Fitting the inertial odometry outputs, namely step length and step heading of a human for instance, with spatial information is an ubiquitous way to correct for the cumulative noises. This so-called map-matching process can be achieved in several ways. In this paper, a novel real-time map-matching approach was developed, using a backtracking particle filter that benefits from the implemented geospatial analysis, which reduces the complexity of spatial queries and provides flexibility in the use of different kinds of spatial constraints. The goal was to generalize the algorithm to permit the use of any kind of odometry data calculated by different sensors and approaches as the input. Further research, development, and comparisons have been done by the easy implementation of different spatial constraints and use cases due to the modular structure. Additionally, a simple map-based optimization using transition areas between floors has been developed. The developed algorithm could achieve accuracies of up to 3 m at approximately the 90th percentile for two different experiments in a complex building structure.

## 1. Introduction

The usage of smartphones for navigation, based on the global navigation satellite system (GNSS), is common in the daily life of people, especially for personal navigation. GNSS is the main technology for outdoor localization. However, heavy attenuation and reflection of the GNSS signals by building structures impede sufficient availability and accuracy in indoor-scenarios [1]. Indoor localization poses further challenges due to more complex layouts and space constraints [2], as well as the need for a higher accuracy [3]. However, Indoor Navigation considering the environment constraints is useful for many different applications, for example, in the context of the Covid-19 pandemic, it can be useful to detect the room a certain person is located in, to ensure that only a maximum number of people are in the room and to track contacts to control the spread of the virus. For an accurate room detection, map information, including room sizes and locations, are necessary.

The inertial sensors of a device can be used to track a user’s position by continuously estimating the displacement from a known location. For instance, pedestrian dead reckoning (PDR) [4,5] is not dependent on infrastructure assistance. However, the IMU’s measurements are noisy, resulting in an accumulating positional drift over time. Therefore, it can only provide reliable position estimation for a limited time, making additional support necessary. Several approaches exist, such as a visual support approach using vision features [6]. Other approaches use point clouds [7,8] from cameras, as well as light detection and range (LIDAR) sensors, that can be combined with the odometer information to correct the drift and to provide more accurate localization estimations. These features are not always available since vision sensors can’t be used at all times. However, once map information is available, map matching is among those additional supports that make the correction influences possible at any time on a respective autonomous position estimator [9]. The information about the environment, such as in the form of floor plans and/or routing graphs, can be combined with the odometry outputs to achieve a more accurate position estimation, without the installation of any additional infrastructure.

Simple point-to-point map matching was among the first computationally efficient and simple approaches. It can be viewed as a simple search problem where location points are matched to the respective floor plans. The disadvantage is its sensitivity to how the path network is digitized [10]. Therefore, the obtained trajectory can be matched with the help of geometry and topology information with trajectory matching methods. In [11], the trajectory of a given user is compared to the geometry of the floor plan. Another approach, where the sequence of the users states of motion (standing, walking, turning, etc.) are compared with a map, has been developed in [12]. The advantage of trajectory matching compared to point-to-point matching is its higher robustness and smaller errors. Its disadvantage, on the other hand, can be its high complexity and possible poor ability for real-time applications [13].

In recent studies, map-matching algorithms based on probabilistic graphical models fit motion trajectories to a floor plan by using conditional random fields [14,15], hidden Markov models [16,17,18,19], a Kalman Filter [9,20], particle filtering (PF) [21,22,23], or dynamic programming [14] in a real-time and sequential manner. In more detail, the probabilities of each location are updated according to spatial constraints, and the state update can be done according to the last steps. Our literature review confirms the superiority of the conditional random fields methodology over other sequential map-matching methods with respect to accuracy and complexity [24], but they usually rely on learning data and the map material needs to be prepared in a specific way [14]. PF can generally perform a robust and real-time map matching by modeling complex sequential motion models, as well as the non-linearity of noises, without any learning of data.

PF belongs to the Monte Carlo Methods, which are groups of state estimation methods used to model highly non-linear problems with very noisy measurements [25]. PF is able to handle highly non-linear problems, where the uncertainties are represented by several estimation samples (so-called particles) [26]. The goal in the implementation for map matching is to approximate the probability distribution function (PDF) of a position by a large number of weighted, independent particles [27]. Backtracking PF further consider the history of the trajectory to further improve position estimates [28].

For this study, a real-time map-matching approach based on a backtracking particle filter was developed. This work is based on insides acquired through the same authors previous publication in the IPIN conference [29]. The focus is on map matching using the same available data set, but with a backtracking PF instead of the bootstrap PF. The novelty of the map-matching approach developed in this paper is the possibility to use any kind of odometry information as an input. This enables its use on all kinds of devices, from robots to wearables to smartphones. This poses an advantage to other common approaches, which are only suited for specific-use cases (such as a smartphone, odometer, and wearable sensors). Further, the implemented geospatial analysis tools helped to reduce the complexity of the spatial queries and aided in the development of the modular and flexible structure of the algorithm. This enables one to easily add, modify, and exchange different spatial constraints for the particle validation, including routing-graph-based concepts, and to adjust the algorithm to different use cases. The modular structure provides flexibility for further research, development, and comparison. Additionally, the use of geospatial analysis tools permits the comparatively easy use of map information from files in the GeoJSON format. The structure of the PF algorithm was a good fit to implement the geospatial analysis tools and realise the modular structure of the map-matching algorithm.

**Figure 1:**
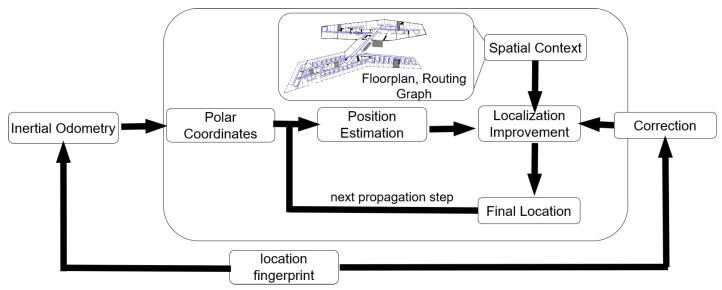
Overview of the structure of the developed algorithm that uses particle filtering as a map-matching process. Starting from a position derived, for example, through a location fingerprint, odometry data from inertial measurements are transferred to polar coordinates, resulting in the estimation of the propagated position. This estimation is improved via spatial constraints and further corrected by additional information from the location fingerprint, resulting in the final position estimate, which serves as the starting point for the next propagation step.

Further, a simple correction mechanism based on so-called location fingerprinting was implemented, enabling the initialization of the start position and further corrections of positions, if further environment-dependent measurement patterns are detected. This can also include environmental signals, such as Fifth-Generation of Mobile Telecommunications Technology (5G) signals or GNSS, as introduced in [29]. In addition to the 5G correction in [29], here, further environmental information including, but not limited to, transition zones between floors (e.g., elevators or stairs) can be considered in combination with height information from barometer measurements. The overall high-level structure of the developed algorithm is shown in Figure 1. The polar coordinates (angle of orientation and traveled distance) used for the position estimation can be derived from different methods, such as PDR or odometry learning approaches. In this work, the focus has been on pedestrian navigation, and, for this reason, the polar coordinates are referred to as step headings (direction of movement as angle of orientation) and step lengths (traveled distance per time as the length of each step).

## 2. Methodology

This section describes and explains the underlying methodologies of this work, including geospatial analysis, the backtracking particle filter, and the corrections based on location fingerprints.

### 2.1. Geospatial Analysis

Geospatial analysis describes the process of extracting, manipulating, and visualizing spatial information [30]. One use case for geospatial analysis is the investigation of the spatial relationship between geometric objects. The most fundamental geometric objects are so-called geometric primitives. These are geometric objects that can be described by one continuous geometry and include points, curves, surfaces, and solids [31].

Geospatial analysis can be done with a variety of tools, including geographic information system (GIS)-software such as QGIS [32] or ArcGIS [33] or by using programming languages, for example Python, with libraries such as GeoPandas [34]. GeoPandas is an open-source project, extending the data types used by pandas to enable spatial analysis on geometric objects. These data types are *GeoSeries* and *GeoDataFrames*. The fundamental geometric objects are implemented in Shapely (as *Point* class), *LineString* class, and *Polygon* class. Several geometries of the same kind can be unified as collections, resulting in so-called *MultiPoints*, *MultiLinestrings*, and *MultiPolygons* [35]. *GeoSeries* and *GeoDataFrames* can store one or several different geometric objects. *GeoDataFrames*, which are basically extensions of the *DataFrames* used in the Python library Pandas, also enable the storage of attributes of geometric objects. This spatial data structure includes spatial relationships between geometric objects, such as contains, intersects, overlaps, touches, etc. Geometric operations, such as the investigations of the mentioned spatial relationships, are performed by Shapely [35]. Queries for spatial relations will return a Boolean value (*True* or *False*). Some queries for spatial relationships, implemented in Shapely, are listed below:

- ***Intersects*** returns *True* if the boundary or interior of the object intersect in any way with those of the other;
- ***Contains*** returns *True* if no points of the other geometry lie in the exterior of this geometry and at least one point of the other geometry’s interior lies in the interior of this geometry. A line’s endpoints are its boundary and are therefore not *contained*;
- ***Within*** returns *True* if this object’s boundary and interior intersect only with the interior of the other (not its boundary or exterior), making it the ‘inverse’ of *contains*;
- ***Touches*** returns *True* if the given objects’ boundaries have at least one point in common, but their interiors do not intersect with any part of the other.

**Figure 2:**
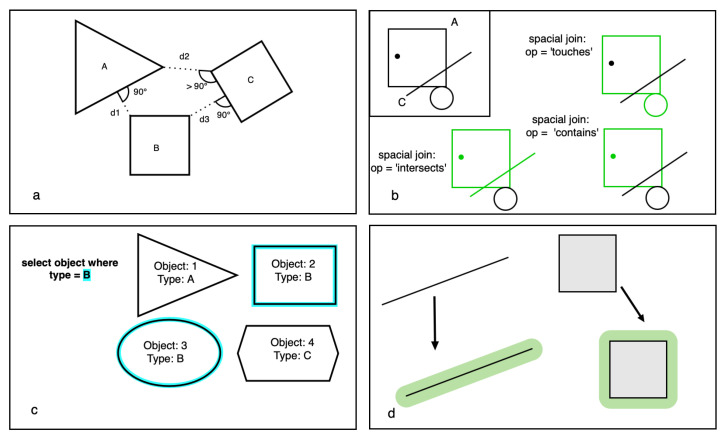
Overview of some of the geospatial analyses used in GeoPandas, with *distance* shown in (**a**), *spatial join* in (**b**), *query by attribute* in (**c**), and *buffer* in (**d**).

The function of some of the fundamental geospatial analyses that can be applied to *GeoSeries* and *GeoDataFrames* are listed below and are shown in Figure 2:

- ***Distance*** returns the direct distance between geometries. It is also the orthogonal distance between a point and a line feature or edge, if the orthogonal projection of the point exists (see Figure 2a). Here, the distance from the corners of polygon B and the edges of the polygons A and C is simultaneously the orthogonal distance, whereas the distance between the corners of A and C is not);
- ***Spatial join*** is used to merge geometric objects based on their spatial relationships, as shown in Figure 2b. Spatial relationships, that can be used as requirements for the spatial join include *intersects*, *contains*, *within*, or *touches*;
- ***Query by attribute*** returns all objects of the given *GeoDataFrame* that hold the queried attribute value, as shown in Figure 2c, where all objects with the *Type* value of B are selected and marked in blue;
- ***Buffer*** generates a representation of all points in a given distance of the geometry as a polygon. In Figure 2d, the resulting buffer-polygons around a given line and a square-shaped polygon are displayed in green.

### 2.2. Backtracking Particle Filter

This section explains the principles of the backtracking PF. The idea of a PF in the application of navigation, specifically for PDR, is to approximate the PDF of the position by a large number of weighted, independent particles [27]. The weighted mean of the particles will then result in a position estimate.

When using a PF for positioning with map information support, paths can be estimated incorrectly. For example, a change in direction occurs at a location where there are several junctions close by. It can happen that the path follows the wrong junction, due to uncertainties in the positioning. The wrong path might lead to an aborting of the filter, due to strong corrections from the map information. If the scattering of the particles is wide enough, part of the particles might travel the correct path. In this case, a backtracking functionality can recalculate the path from the stored history of the movements of the particles.

In the following, such a backtracking PF, a variation of the PF model, is explained in detail. Many other variations of the PF exist, which mainly differ from each other by modifications of one or more of the components. For examples of other PF modifications, see [26,36], among others. A PF with backtracking works in its basic functionality similar to a simple bootstrap PF. However, compared to the bootstrap PF, it also considers the particle trajectory’s history to improve position estimates. As in the bootstrap PF, at first, a set of initial particles is created, representing the PDF of the starting position. The starting position can either be known or unknown. For the PDR, the particles are either all located at the known starting point, as in [9], or they can be distributed around the starting point according to the uncertainty of the position. In other cases, for example in combination with absolute positioning methods such as received signal strength indication RSSI, the particles could also be randomly distributed over the whole area, as done by [37]. The backtracking PF then consists of three steps: the propagation, filtering, and backtracking.

During the propagation step, the individual particles are changed to better represent the PDF. In pedestrian positioning, the particles can be spatially propagated according to the length and heading of the current step, which are derived from the odometry outputs. Possible measurement errors can be taken into consideration by assigning a randomly distributed noise to step length and step heading for each particle, as done by [9]. After the propagation, the trajectories of invalid particles, for example those that cross walls or non-accessible areas, are deleted during the so-called filtering step. Other approaches can be used, filtering the particles based on the distance to routing lines or based on the room that they are located in.

After the deletion of invalid particles in the filtering step, the position estimate is calculated from the mean of the particle positions and the orientation of the device is updated by the mean of the products of each particle’s noise value for the orientation and the measured orientation for the position. Optionally, the remaining valid particles can also be weighted according to spatial constraints, for example their distance to the closest routing edge. The weighted mean of these particles results then in the position estimate.

**Figure 3:**
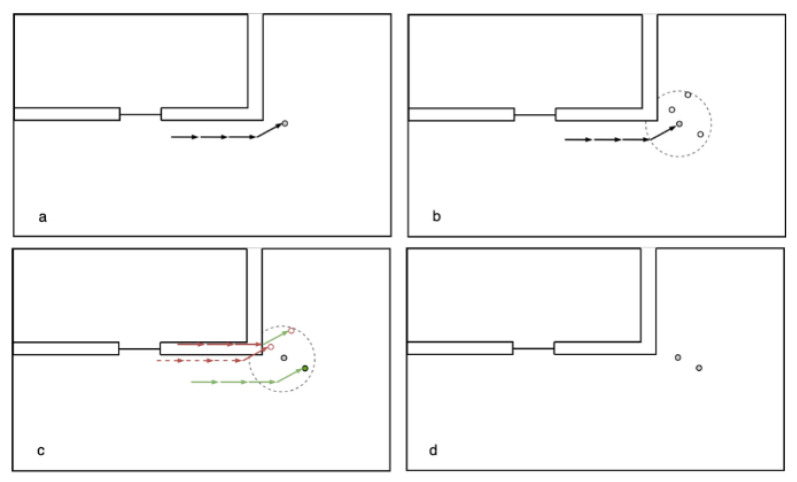
Overview of the backtracking process. A particle with valid trajectory is randomly chosen (**a**). Particles are randomly created in a specified radius (**b**). Newly created particles with invalid trajectories (red) are discarded until a particle with a valid trajectory (green) is found (**c**) and is accepted as a new particle (**d**).

Next, for every particle that was deleted in the filter step, a new particle attempts to be generated within a specified and random radius around a valid particle. The potential new particle’s propagation history, according to the displacement of the steps, is then checked for their validity according to the above-mentioned filter methods. If the trajectory is valid, the particle is accepted, otherwise a particle with a different position and a different random scale for the displacement is proposed, as shown in Figure 3. During the backtracking segment of the PF, new particles are generated until the maximum number of particles is reached again.

### 2.3. Location Fingerprint

The location fingerprinting is a similar concept to the landmarks approach in map matching [13]. Contrary to other landmark approaches, where a dedicated graph of the location of specific features is created, here, only isolated information is used when possible to correct the position of particles. This can basically be any information that is detectable by the used device and is specific to a certain space. This could, for example, include position information from cellular networks, e.g., from a 5G campus network, as implemented in [29]. Another option can be to use the barometer in a smartphone to detect significant height changes that indicate that a user is using stairs or elevators and can, therefore, only be located in such.

## 3. Map Matching Based on a Backtracking Particle Filter

**Figure 4:**
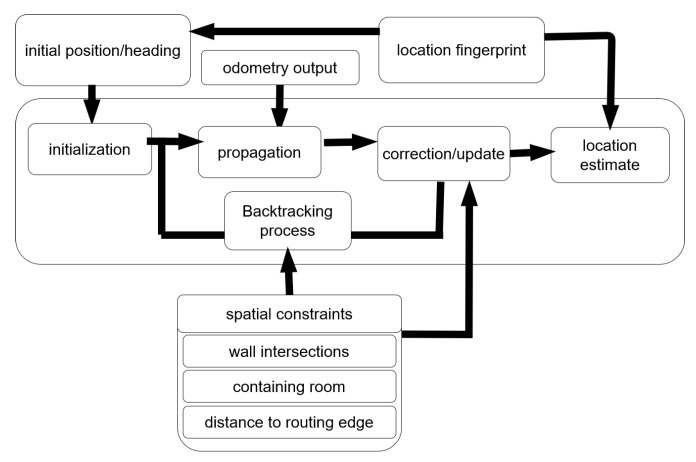
Overview of the particle filter algorithm. The initial position and heading, derived from the location fingerprint, is used for the initialization of the particles, which are then propagated according to the odometry output. The particles are then filtered in the correction/update according to spatial constraints, leading to a final location estimation, which is further improved by the location fingerprint. Via the backtracking process, the number of valid particles is increased up to the original amount of particles by considering spatial constraints. Next, the next propagation step starts.

This section explains the implementation of the developed backtracking PF with geospatial analysis in the map-matching process. In Figure 4, a high-level overview of the developed map-matching process is presented.

The algorithm for the map matching is shown in Algorithm 1 as pseudo code. As input, it requires the odometry data, i.e., the step heading, step length, and step height, as well as the map information (FloorData). In line 2, the initial particles at the start position are created before the *for*-loop from line 3 on iterates over all steps while performing the map-matching processes. For every step, the current floor is determined and the floor data is selected accordingly (line 4 to 6).
**Algorithm 1** Map-matching algorithm with backtracking PFInput: Startpoint,StepHeading,StepLength,StepHeight,FloorDataOutput: result1:maxParticleNumber = 2002:particles←InitializeParticles*(Start,maxParticleNumber,σH,σS)*3:**for***i* in StepNumber **do**4:    CurrentFloor←CheckFloor(*Height*)5:    FloorChanged←True/False6:    set FloorData according to the CurrentFloor7:    Step←Step *(StepLength,StepHeading,Height,CurrentFloor,CurrentConstraints, CurrentPosition,StepScale)*8:    PropagatedParticles←CheckParticleSteps*(particles,Step,FloorData)*9:     weights←WeightingMethod*(PropagatedParticles, FloorData)*10:   PositionEstimate←weightedmeanofparticlespositions11:    append PositionEstimate to Trajectory12:    NumberOfNewParticles←maxParticleNumber−                          number of PropagatedParticles13:    particles←BackTracking*(NumberOfNewParticles,PropagatedParticles,*                         BackTrackingSteps,FloorData,S)14:    **return** PositionEstimate15:**return** Trajectory

Next, a step-object (Step) is created (line 7), which stores the length, heading, height, current floor, the current position estimate, and the room it is located in, as well as all specific spatial constraints for this step (e.g., rooms where particles are allowed) for the current step. In line 8, the particles’ positions are then propagated based on the step length and step heading and are filtered regarding their plausibility according to the chosen filter concept. If the support through additional particle weights is active, the particles’ weights are determined according to the weighting method afterwards, otherwise all weights are set to one. The weighted mean of the particles’ position then provides the position estimate. Following the estimation of the position, the backtracking algorithm (Algorithm 2) is executed to create new particles and add them to the *GeoDataFrame* containing the propagated, valid particles. These are then used in the next loop of the backtracking PF algorithm. After the PF is performed on the last backtracking step, all calculated positions, as well as the standard deviation of each position estimate, is returned and saved as a comma separated values (CSV) file.

In this work, the initial particles are normally distributed around the starting position with a standard deviation of the uncertainty of the start position. The start position can be estimated from the location fingerprint, but it can also be set manually if a location fingerprint is not available. The initial heading of the user can be derived from the angle of the line fitted through the first few positions after the user starts to move in a chosen direction.
**Algorithm 2** Backtracking AlgorithmInput: particle,Steps,ValidRoomsOutput: particles1:Samples←randomChoice*(particles,NumberOfNewParticles)*2:**for***p* in Samples **do**3:    **while** Try>0 **do**4:        NewParticle←ProposeParticle*(p,Radius)*5:        **if** *BackTrackingTest(NewParticle)* is passed **then**6:                 append NewParticle to particles7:        Try=Try−1 **return**  particles

In the backtracking PF with geospatial analysis, the propagation of the particles is integrated in the filter process, in which the particles are checked for their plausibility by a specific concept. In this work, either the checking for line of sight (cl), checking for containing rooms (cm), or checking for distance to routing graph (cr) concept are used (see Figure 3), but other approaches could be implemented as well. The new position of each particle, $X_{i},Y_{i}$, is calculated in the propagation step (see Figure 2) with Equations (1) and (2):

(1) $$X_{i}=X_{last,i}+\left(S+\epsilon_{l,i}\right)*cos\left(H_{0}+H_{gy}+\epsilon_{h,i}\right)$$

(2) $$Y_{i}=Y_{last,i}+\left(S+\epsilon_{l,i}\right)*sin\left(H_{0}+H_{gy}+\epsilon_{h,i}\right)$$

where $H_{0}$ is the initial heading, $H_{gy}$ is the estimated heading from the odometry data, *S* is the measured step length, and $\epsilon_{l,i}$ and $\epsilon_{h,i}$ are the noise values for the step length and step heading for each *i*th particle, respectively.

**Figure 5:**
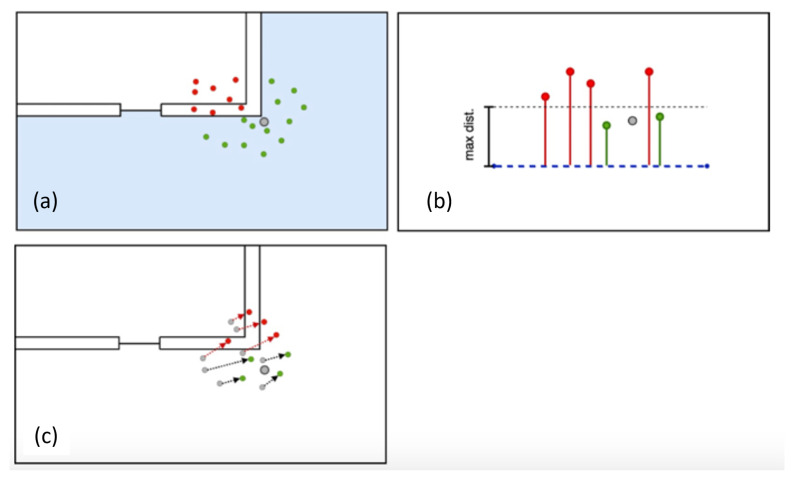
Overview of the used filtering methods, with the cm in (**a**), cr in (**b**), and cl in (**c**).

For this paper, three filtering methods for the validity of particles have been used, namely the checking by line of sight (cl), checking by rooms (cm), and checking by distance to the routing graph (cr) concept. For the cl (see Figure 3c), a GeoDataFrame is created containing all the connections between each propagated particle and its previous position. Every particle whose corresponding connection line intersects a wall is deleted, while all other particles are kept in the *GeoDataFrame*. Finally, the *GeoDataFrame* with the remaining particles is returned. The cm (see Figure 3a) concept is similar to cl. However, here, the walls are not required as map information, but the rooms, considered as valid for the particles (ValidRooms), and doors. For each propagated particle, it is checked if it is either within one of the valid rooms or if the *LineString*-geometry from its new and its previous position intersects either with any door-*polygon* or any *polygon* contained in Transitions. If that is the case, it is appended to the valid particles, which are finally returned as a *GeoDataFrame*. Rooms, which are entered by particles through doors are added to the list of valid rooms and kept valid as long as particles are inside them. In the cr concept (shown in Figure 5b), for each propagated particle, the smallest distance to the routing edges is determined. Only if the smallest distance to the routing edges for a particle is smaller than 2 m, is it then appended to the list of valid particles. The valid particles are then, as for the other concepts, returned as a *GeoDataFrame*.

The correction concept is implemented in the algorithms of the explained filtering methods. In this work, the location fingerprinting is based on floor changes. After the propagation of the particles, the height change over a defined number of recent steps is observed. If this height change exceeds a specified threshold, all particles that are not located in transition zones (stairs and lifts) connecting the current and the new floor are deleted. The remaining particles are then filtered according to the implemented filtering methods.

The underlying algorithm of the backtracking segment is presented in Algorithm 2. First, a number of particles, according to the difference between the number of valid particles and the maximum number of particles, is randomly chosen from the currently existing particles (line 2). For each sample particle, a new particle at a random position inside a specified radius (line 1) is proposed (line 5). For this proposed particle, a backtracking test (see Algorithm 3) is performed and, if it passes the test, it is appended to the existing particles (line 6 to 8). If the particle doesn’t pass the backtracking test, the procedure is repeated until either the maximum number of particles is reached or until a maximum number of tries (in this case eight tries) has been reached. The limited number of tries will prevent an unnecessary slowing down of the algorithm due to a very high number of tries for each particle. The *GeoDataFrame*, to which the accepted particles are added, is finally returned.
**Algorithm 3** Backtracking testInput: particle,Steps,FloorDataOutput: True/False1:**for** each *s* in Steps **do**2:    *propagate(particle,s)*3:    **if** *CheckValidity(particle,FloorData)*←*valid* **then**4:        continue with test5:    **else**6:        **return** False  **return** True

For each concept used for the checking of particles, the same concept is used for the backtracking test. For each backtracking step i−1, backwards from the current step *i*, the new position $X_{p,i-1},Y_{p,i-1}$ of each particle *p* is calculated from the current position of the particle $X_{p,i},Y_{p,i}$ with Equations (3) and (4):

(3) $$X_{p,i-1}=X_{p,i}-\left(S_{i-1}+\epsilon_{l,p}\right)*cos\left(H_{0}+H_{gy,i-1}+\epsilon_{h,p}\right)$$

(4) $$Y_{p,i-1}=Y_{p,i}-\left(S_{i-1}+\epsilon_{l,p}\right)*sin\left(H_{0}+H_{gy,i-1}+\epsilon_{h,p}\right)$$

where $S_{i-1}$ is the step length of the previous step and $H_{gy,i-1}$ is the heading of the previous step. Each backtracking step is then checked for its validity (intersections with walls for the cl, containing rooms for the cm, or distance to routing edges for cr). If the whole trajectory passes the backtracking test, the algorithm returns *True*, otherwise the algorithm returns *False*. When the cl concept is used, it is not checked for intersection at every step, but for all steps simultaneously because the query for intersections is the most time-consuming step and the usage of *GeoDataFrames* makes it more efficient to query for intersections of the *LineString*-geometry representing the whole trajectory than to query for intersections at each step if multiple steps are considered.

## 4. Test Data

The map matching based on the backtracking PF has been tested with the three different concepts, cl, cm, and cr, for the particle checks on two different trajectories. For the two test trajectories, the step length and orientation from PDR based on the smart phone’s INS was used. The used data sets, including the building information and the data for step heading, step length, and height is described in this section. The data has been provided by [29]. The sensor data as well as the floor plans are available on the GitHub platform, linked in the data availability statement section at the end of the article.

### 4.1. Building Information

**Figure 6:**
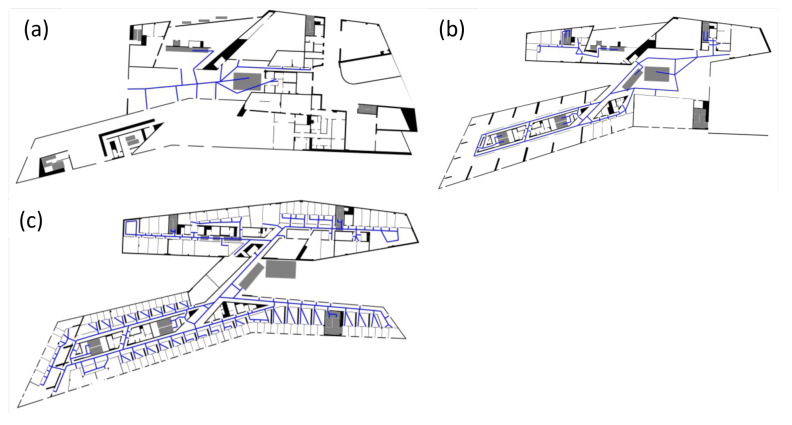
Floor plans of the ground floor (**a**), 1st floor (**b**), and 4th floor (**c**), with the blue lines representing the routing edges.

The building information of the HafenCity University Hamburg (HCU) has been provided as floor plans in GeoJSON format, that was extracted from CAD plans [29] and have been minimally cleaned up manually. They contain polygons with attributes according to their type (e.g., walls, rooms, hallways, staircase, etc.). The floor plans have been provided separately for each, the ground floor, 1st floor, and 4th floor. The floor plans are loaded in the developed algorithm in *GeoDataFrame* structures, handled by the *GeoPandas* library. The complex architecture of the HCU building, including big, open hallways, small corridors, as well as unconventionally shaped rooms with pointy corners (see Figure 6), results in a distinct environment to test the developed PF under various conditions.

### 4.2. Sensor Data

The values for the step length and the step heading for the two tested trajectories, which have been estimated during a previous study of the authors [29], have been readily provided in the CSV format. The provided step headings are the orientation deviations in relation to the orientation at the starting point, making it necessary to add the absolute starting orientation to each heading value to derive the absolute heading to geographic north. The model for the step length calculation from the IMU values were based on a person with a different height and, therefore, a different step length than the persons who were generating the test data, which leads to a significant systematic error for the step length values. This provided a situation in which the robustness of the map-matching algorithm to systematic errors of the step length could be evaluated. The height difference for each step was also provided as a CSV file. To get the absolute height at any given step, the height differences until that step had to be summed up and added to the starting height. The starting height was approximated as the height of the starting floor plus 80% of the users’ height. The ground truth of the walked trajectories has been provided as a CSV-file as well and contains the horizontal position, time stamp, and the height difference for each point.

**Figure 7:**
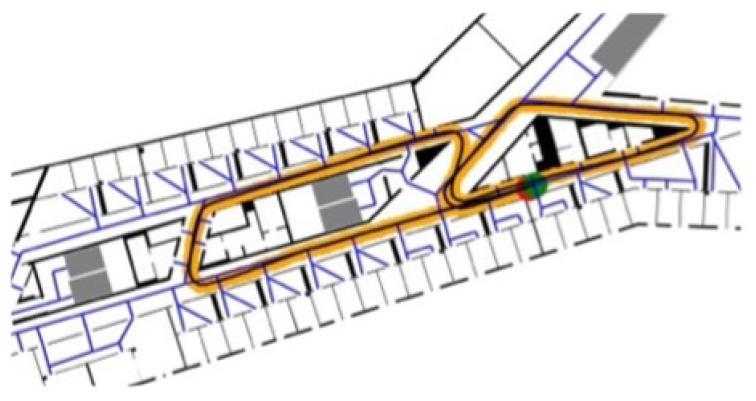
Ground truth points (orange) of the “eight path”; the red dot is the starting position and the green dot is the finish.

The height of the floors was estimated as 0 m for the ground floor, 6 m for the 1stfloor, and 19 m for the 4th floor. The two trajectories are referred to as *eight* path and *zerotofour* path in the rest of this thesis. The *eight* path starts in a corridor of the 4th floor in front of an office in the western direction and continues in the shape of an eight (see Figure 7 for the ground truth of the *eight* path).

**Figure 8:**
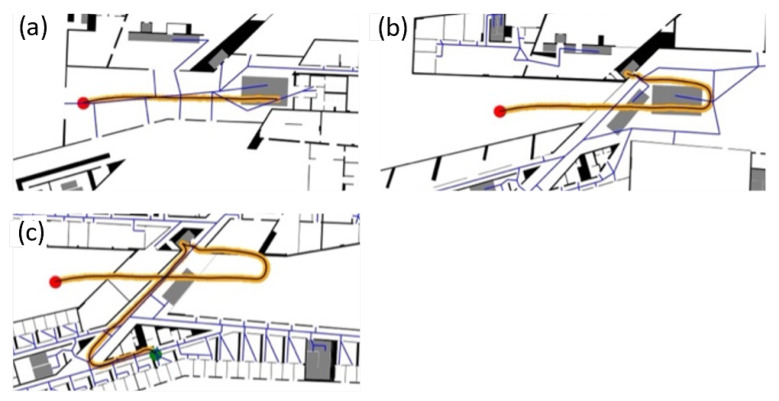
Ground truth points (orange) of the *zerotofour* path in (**a**–**c**); the red dot is the starting position and the green dot is the finish.

The *zerotofour* path starts in the entrance hallway on the ground floor (Figure 8a) and leads over the main stairs to the 1st floor. It than takes a left turn and proceeds to take the elevator figure (Figure 8b) to the 4th floor. It continues along the corridor in the 4th floor southwards, then takes a turn at the t-junction and leads into an office room in the south-eastern part of the 4th floor (Figure 8c).

## 5. Results

**Figure 9:**
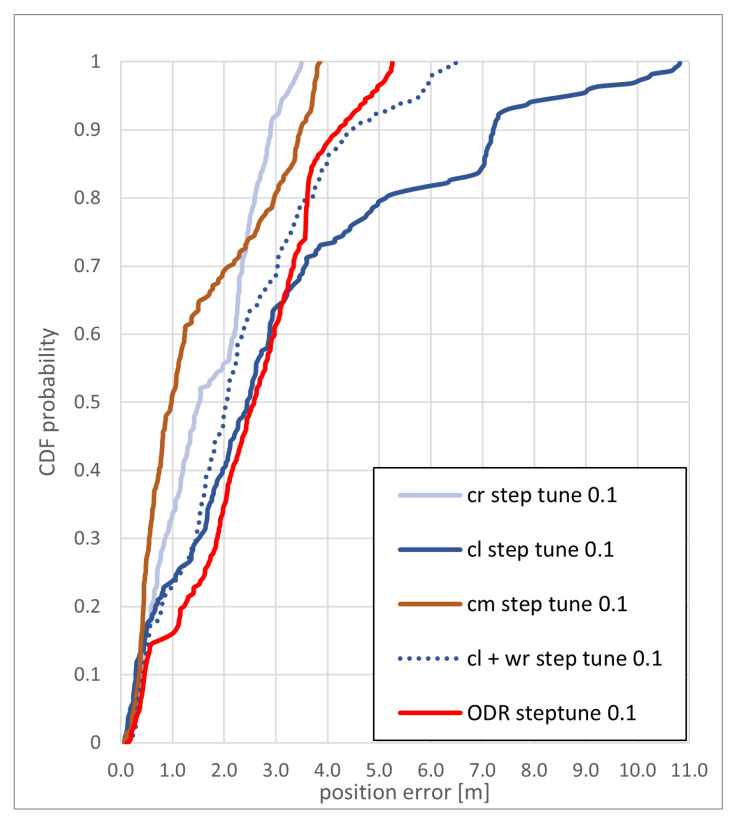
CDF of the positioning error of the *eight* path with a step length correction of 0.1 m for the backtracking PF and the PDR with a step length correction of 0.1 m.

In this section, the results of the map-matching algorithm using the cr, cm, and cl method for particle checks are presented and discussed for the two test trajectories. The cumulative distribution function (CDF) of the position estimate error for the different concepts used for the *eight* path, with a step correction (manual step scale) of 0.1 m, is shown in Figure 9. The cm and cr method reached an overall similar accuracy, with the cm method reaching an accuracy of less than 4 m and the cr method reaching a accuracy of just less than 3 m in 90% of the steps.

**Figure 10:**
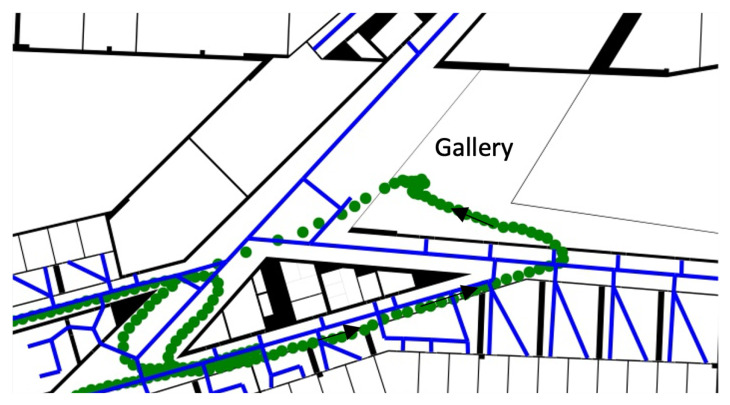
Part of the trajectory of the *eight* path, that wrongly proceeds in the “gallery”, when using the weighting by routing support method, where black arrows indicate the walking direction and the green points represent the position estimates for each step.

The cl method performed significantly worse; this is mainly due to the situation that at a step correction of 0.1 m, enough particles are overshooting at one of the turns, ending up in the “gallery” that the positions for the next steps is estimated to be on the wrong side of the wall (see Figure 10).

**Figure 11:**
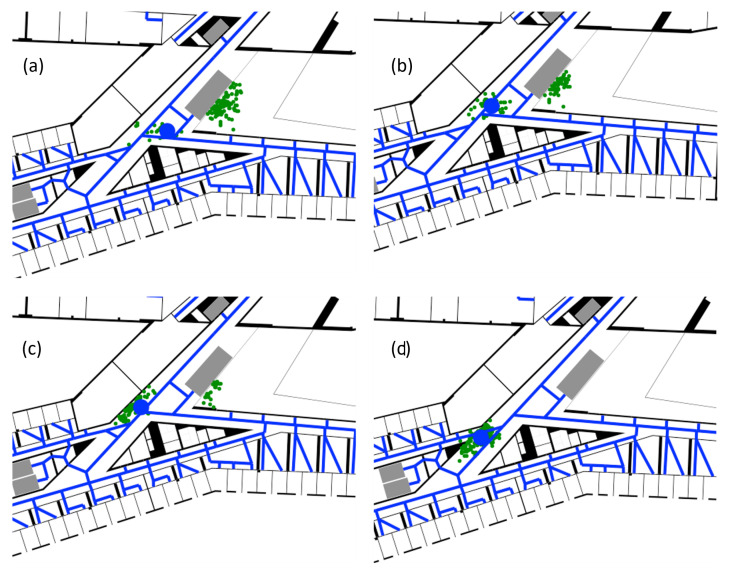
Example of the trajectory correction (from (**a**–**d**)) through the backtracking functionality, where the green dots represent the valid, propagated particles, and the blue dot is the resulting position estimate. First, most particles took a “wrong” path (**a**), continuously getting deleted when trying to pass the wall (**a**–**c**). While more particles are getting deleted, more particles with plausible trajectories are getting created through the backtracking process, until all particles are recovered and now only represent positions with reasonable trajectories again (**d**).

At this point, the backtracking functionality can be observed (see Figure 11). At the end of the “gallery”, with every subsequent step, more particles get deleted because they would cross a wall, resulting in the backtracking algorithm resampling the invalid particles at more plausible positions and by this, changing the trajectory to the correct path.

**Figure 12:**
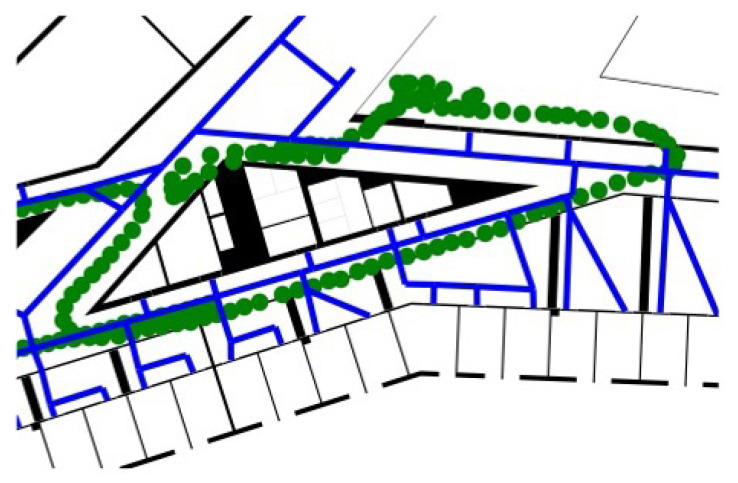
Effect of the support through the weighting by routing support on the deviated trajectory.

This was also an exception, where the weighting of the particles corresponding to the distance to the nearest routing edge helped to significantly increase the accuracy to less than 4.5 m in 90% of steps, by correcting the trajectory closer to the next routing edge (see Figure 12). Since the “gallery” does not contain any routing edges, the trajectory was directed back into the corridor. The effect of the weighting method was negligible for the other methods for the tested trajectories.

**Figure 13:**
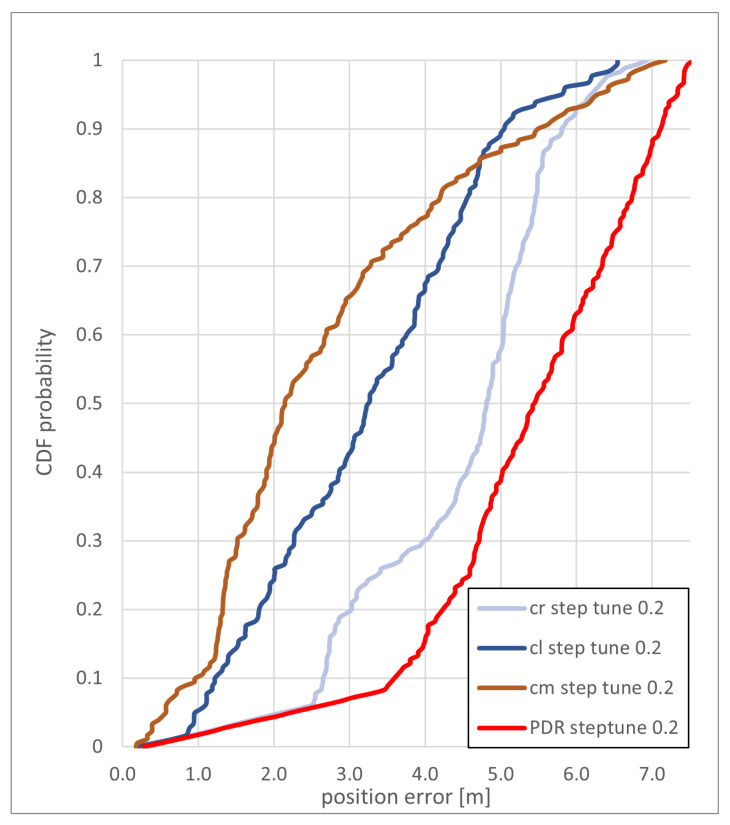
CDF of the positioning error of the *zerotofour* path with a step length correction of 0.2 m for the backtracking PF and the PDR with a step length correction of 0.2 m.

Figure 13 shows the CDF of the position estimate error for the *zerotofour* path with a step correction of 0.2 m. The accuracy for all methods is between 5 and 6 m in 90% of the steps. The cm method can provide a position error of less than 4 m more often than the two other methods. From the three methods, only the cm method lead the trajectory into the elevator, while only the cr method reached the final room. The cm and the cl method both lead the trajectory into the neighboring room, west of the final room.

**Figure 14:**
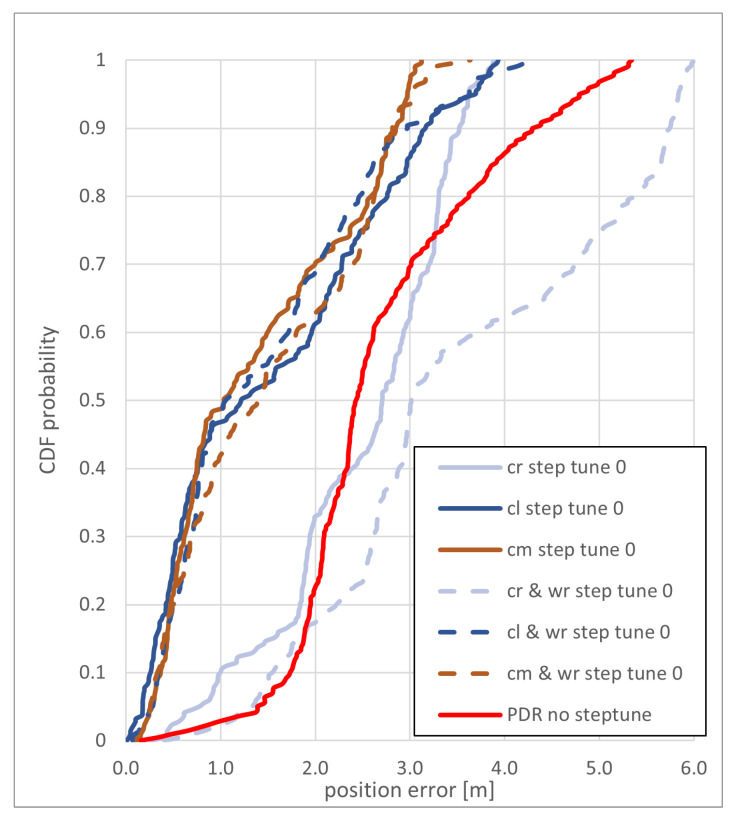
CDF of the positioning error of the *eight* path without step length correction for the backtracking PF and the PDR without step length correction.

**Figure 15:**
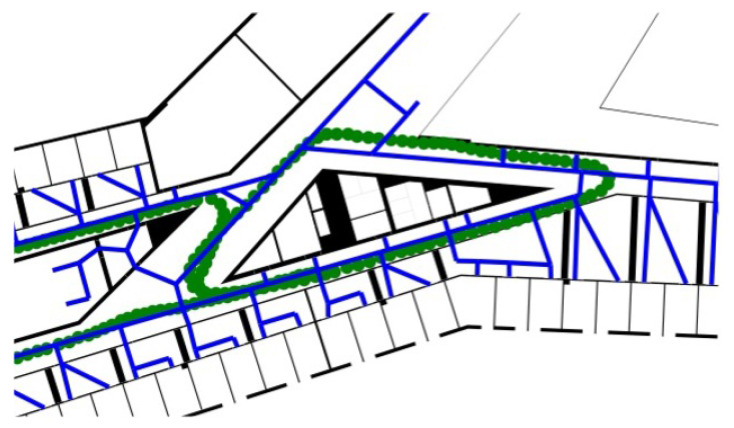
Trajectory (green dots) from the cl method for the *eight* path without step length correction for the backtracking PF.

For the *eight* path without step correction, an accuracy better than 3 m can be achieved with the cm and cl method, while the overall performance of the cr method falls behind the two other methods, as shown in Figure 14. The use of additional routing support worsens the achievable accuracy of the cr method. For the cm and cl method, the weighting by the distance to the routing graph has only a small influence on the error of position estimate. The cl method performs significantly better (also see Figure 15), mainly because there is less particle overshoot at the turn at the eastern corner. This way, the trajectory doesn’t deviate into the “gallery”.

**Figure 16:**
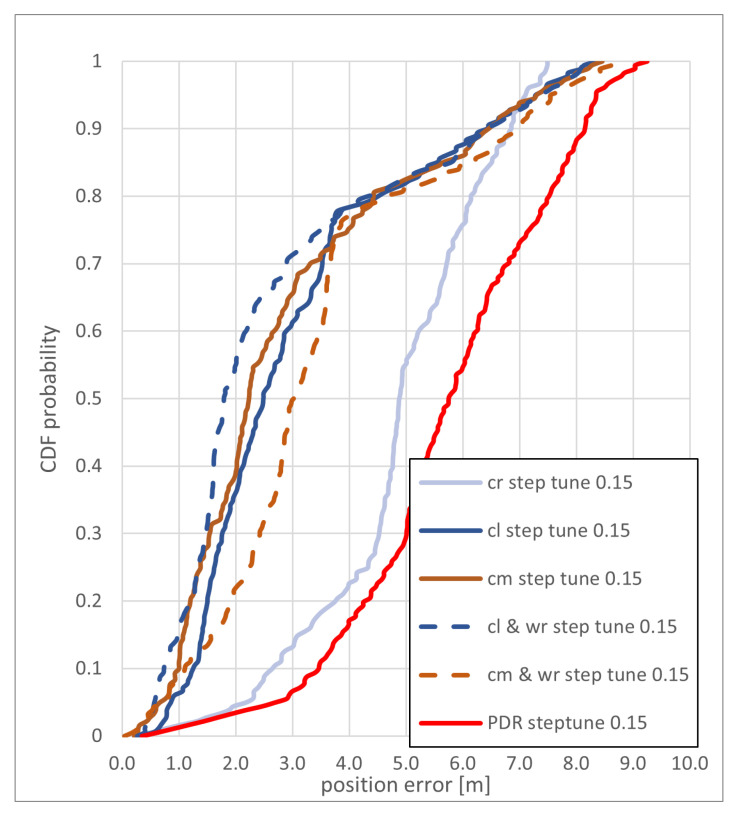
CDF of the positioning error of the *zerotofour* path with a step length correction of 0.15 m for the backtracking PF and the PDR with a step length correction of 0.15 m.

Figure 16 shows the different method’s CDF of the positioning error for the *zerotofour* path with a step length correction of 0.15 m. All methods, with and without the additional use of the weighting method, achieve a positioning error of less than 7 m in 90% of the steps, while the cm and cl methods achieve a significantly better accuracy for the positioning than the cr method. The additional weighting of particles only has a minor influence on the accuracy of the used methods, slightly improving the cl method and slightly decreasing the accuracy of the cm method. The elevator could be reached by the cl method as well as the cl and cm methods with routing support. It was closely missed by the cm method. The cr method was the only one that reached the destination room, with all the other trajectories ending in the same neighboring room.

In most cases, the cm and the cl method had the best accuracy. The *eight* path with a step correction of 0.1 m is the exception; here, the cl method reached a significantly worse accuracy, due to the above mentioned situation in which the trajectory gets “trapped” in the gallery for a part of the trajectory.

## 6. Conclusions

Fitting the odometry outputs with spatial information is a way to correct for the cumulative noises. In this paper, a real-time map-matching method based on a backtracking PF has been developed that can handle different odometry inputs. It provides flexibility to use different kinds of spatial constraints due to the usage of geospatial analysis tools and can be implemented in different use cases. We could successfully develop a PF algorithm that autonomously supports a meter scale position accuracy for indoor navigation. We took the benefits of both odomotery and geospatial anaylsis to develop and evaluate possible algorithms that can provide map-matching services for any kind of moving object. For this, first the underlying concepts and methodology were explained and related work was presented. Next, the concepts as they were further developed and adjusted for this paper have been explained, including the detailed explanation of the implementation of the algorithms of the backtracking PF. Finally, the used test data set was presented and the results of the backtracking PF with geospatial analysis have been investigated and discussed. In addition, a simple correction method has been developed and implemented that can use additional environmental information to further improve the position estimate. In this paper, the height information from a smartphone was used to determine if a user is located on stairs or in elevators in order to develop a simple corrector/optimizer by these transition zones. An extended optimization can be done with other position information, e.g., cellular positioning such as 5G, for a continuous correction. Three methods were developed that determine invalid particles based on different building information, namely the cl, cm, and cr method. The results of the different methods have been tested with the data from two paths through the HCU building and have been compared with regard to the attainable position accuracy, as well as the ability to detect certain rooms.

The cm and cl methods reached better precision than the cr method, with the cm method being a bit more robust. It was possible to develop a PF algorithm that could reach an accuracy of 2 to 7 m most of the time in the two tested trajectories. Of all the tested methods, the cm method for particle checking might be favorable, not only because its performance regarding the position precision and room accuracy, but also because additional information for the accessibility of rooms can be relatively easily implemented. n broken elevator could, for example, easily be declared as an invalid space.

Due to the simple and modular code structure, it is possible to add or exchange methods and functionalities to the developed map-matching algorithm. This would also include the additional use of position estimation, for example from 5G antennas, for the correction method. Since polar coordinates are used for the propagation model, in this case, step length and step heading, the usage is not limited to pedestrian navigation, but the developed approach could also be implemented for other use cases such as the indoor navigation of robots or any other moving object, as long as the traveled distance (which can easily be calculated from the speed) and moving direction is provided. Compared to conditional random fields, a PF does not need learning data, making it more convenient and versatile to use in a variety of real-life scenarios. To improve the accuracy in situations where large hallways provide only few possibilities for map corrections, position information from cellular networks (e.g., 5G) could be used in the implemented correction approach to improve the accuracy (see [29]).

The accuracy of the developed PF approaches is dependent on the quality of the provided map information, resulting in better performances with better map materials. The quality of the step length estimation could be improved either by scaling the step length every time an absolute position can be attained with high precision and accuracy, either through infrastructure support or when a position is determined with relatively high confidence regarding their correctness; for example, when using an elevator changes the floor. This could be implemented during the implemented correction method. On the other hand, machine-learning algorithms could be used for a more precise step length estimation. Another option would be to use data sets of step length in relation to other biometric information from a huge number of people. However, these are costly and hard to attain. Overall, a novel map-matching algorithm, based on a backtracking PF, has been developed that provides a meter scale accuracy and is applicable in a large variety of use cases and can easily be modified to handle different kinds of spatial information in different scenarios.

## Data Availability

The desired data is available on the GitHub platform, with the same data-set as in the IPIN conference publication: https://github.com/Hossein-Shoushtari/IPIN21Data (accessed on 15 February 2022).
